# Diversity is maintained by seasonal variation in species abundance

**DOI:** 10.1186/1741-7007-11-98

**Published:** 2013-09-04

**Authors:** Hideyasu Shimadzu, Maria Dornelas, Peter A Henderson, Anne E Magurran

**Affiliations:** 1 Centre for Biological Diversity and Scottish Oceans Institute, School of Biology, University of St Andrews, St Andrews, Fife KY16 9TH, UK; 2 Department of Mathematics, Keio University, 3-14-1 Hiyoshi Kohoku, Yokohama 223-8522, Japan; 3 CESAM, Department of Biology, Universidade de Aveiro, Aveiro, Portugal; 4 Pisces Conservation, IRC House, The Square, Pennington, Lymington, Hants SO41 8GN, UK

**Keywords:** Species coexistence, Biodiversity, Fluctuation mediated coexistence, Storage effect, Stability

## Abstract

**Background:**

Some of the most marked temporal fluctuations in species abundances are linked to seasons. In theory, multispecies assemblages can persist if species use shared resources at different times, thereby minimizing interspecific competition. However, there is scant empirical evidence supporting these predictions and, to the best of our knowledge, seasonal variation has never been explored in the context of fluctuation-mediated coexistence.

**Results:**

Using an exceptionally well-documented estuarine fish assemblage, sampled monthly for over 30 years, we show that temporal shifts in species abundances underpin species coexistence. Species fall into distinct seasonal groups, within which spatial resource use is more heterogeneous than would be expected by chance at those times when competition for food is most intense. We also detect seasonal variation in the richness and evenness of the community, again linked to shifts in resource availability.

**Conclusions:**

These results reveal that spatiotemporal shifts in community composition minimize competitive interactions and help stabilize total abundance.

## Background

Seasonal variation in the abundances of plants and animals will have been apparent to our earliest ancestors, but Gilbert White’s 1789 [1] account of the annual arrival of swifts at the church tower in Selborne is probably the first systematic record of seasonal change in a natural population. Today, most of the focus on seasonal variation in species relates to phenology, particularly in the context of climate change (see, for example, [2,3]). However, fluctuations in abundance have long been hypothesized to affect species interactions in ways that promote coexistence. Here we show that seasonal fluctuations underpin the maintenance of diversity in a multispecies community.

Explaining how multispecies communities persist [4-7] remains a major challenge in ecology [8-10]. There is a long history behind the idea that temporal variation in environmental conditions and species abundance enables species to coexist. It was for example discussed by Hutchinson [11] as a phenomenon that might help resolve the paradox of the plankton. Temporal variation is also inherent in the intermediate disturbance hypothesis, which proposes that competitive exclusion can be constantly postponed by disturbance [12]. Although it is now clear that the intermediate disturbance hypothesis has little empirical and theoretical support [13], there are mechanisms by which species can coexist through temporal niche partitioning. These are known as fluctuation dependent mechanisms of coexistence (FMC) [14], and predict the conditions under which multispecies communities can stably persist.

There are two main types of FMC: relative non-linearity of competition and the storage effect [14]. The first of these is linked to how different species respond to fluctuations in limiting resources. Under certain conditions, non-linear responses to resource availability allow the coexistence of more species than resources [15]. Non-linearity can arise, for example, from satiation or prey handling time limiting resource uptake at high resource abundance. Asymptotic resource acquisition curves are common in ecological contexts [16]. Non-linearity can lead to stable coexistence of more species than limiting resources because variance and covariance of resource abundance act as additional ‘resources’ [17]. Moreover, abundance fluctuations caused strictly by competition dynamics in a constant environment can also allow the coexistence of more species than resources [18].

The storage effect hinges on three conditions: (1) species have different responses to the environment; (2) there is covariance between the environment and competition; and (3) life history buffers population dynamics via seed banks, larval stages or long lifespans for example [19]. These three ingredients combined allow stable coexistence by maximizing intraspecific competition relative to interspecific competition at high abundances, and protecting species from extinction at low abundances [14]. The storage effect results in temporal niche differentiation: species diverge in terms of when they use resources, instead of which resources they use. The two types of FMC are not mutually exclusive: it is likely that both non-linear relationships between resource abundance and population growth rate, and covariance between environment and competition co-occur in ecological communities. Both types of FMC predict asynchronous fluctuations of competing species.

Asynchrony in fluctuations of species abundances is also key for ecosystem stability. The diversity-stability debate has its roots in the discovery that, contrary to conventional wisdom [20], populations in model ecosystems become less stable as diversity increases [5]. However, empirical studies typically suggest a stabilizing effect of diversity (see, for example, [21]). A critical insight was that diversity may increase variance of populations, but it decreases the temporal variance of ecosystem properties (a metric of ecosystem stability) [22]. This is only true if species fluctuate asynchronously so that fluctuations cancel each other at the aggregate scale (synchronous fluctuations have the opposite effect of amplifying variance at the aggregate scale). Ecosystem stability arises from the portfolio effect [23]: independent or negatively correlated temporal fluctuations in species abundances dampen fluctuations of aggregated abundances. Moreover, asynchrony in responses to environmental fluctuations is one of the key reasons diversity protects and enhances ecosystem productivity (the insurance hypothesis [24]).

Quantifying asynchrony in fluctuations of species abundances is challenging for at least three reasons. First, it is a data-intensive exercise because it requires long-term, high-resolution time series of species abundances. Second, there are numerous (often conflicting) processes that contribute to variation in species abundances, including environmental conditions, resource availability and interactions between species (competition and predation) as well as demographic stochasticity. Disentangling the contributions of these different sources of temporal change is a key challenge in the analysis of biodiversity time series [25]. Third, in all but the simplest communities, quantifying asynchrony is a high-dimensional problem because of the number of pairwise interactions between species and with the environment. Hence, despite seasonal fluctuations in species abundances being obvious even to distracted observers, their contribution to species coexistence (via FMC) and ecosystem stability are poorly understood.

Temporal niche partitioning has been examined in a variety of taxa, including grasses [26], desert plants [27,28], and zooplankton [29] at the annual scale. Seasonal fluctuations in species abundance (see, for example, [30-32]) are one way in which communities change through time but the consequences of this seasonal variation for species coexistence are scarcely documented. People living in temperate climates think in terms of four seasons but in other parts of the world there may be fewer or more seasons, and seasons can reflect changes in rainfall as well as temperature and food availability. Aquatic systems do not necessarily mirror the seasonality seen on land. Thus, while seasonality is likely to have an important impact on diversity, its influence is not necessarily straightforward. To the best of our knowledge, there are no clear empirical demonstrations of seasonal fluctuations contributing to FMC. As we will show there is compelling evidence that these seasonal fluctuations underpin the maintenance of diversity in a multispecies estuarine fish community. Fish communities are the most species rich vertebrate communities and typically have a large fraction of generalist species with resulting high potential for resource competition.

In the present work, we test the hypothesis that similar species fluctuate asynchronously with the seasons. To do so, we examine the temporal patterns of numerical abundance of the 45 core fish species (that is those species that occur in the majority of years) in a 31-year dataset, sampled monthly at Hinkley Point, in the Bristol Channel. To do this we first fit a generalized additive model (GAM) to each core species abundance, and decompose the time series into seasonal and non-seasonal components. Then, using cluster analysis, we identify groups of species that are temporally segregated, and compare the resource use of the species that compose each cluster. We next test the prediction that temporal groups are further segregated in how they exploit the spatial habitat when resource competition is most intense. We use the spatial guilds that the fishes belong to as a proxy for traits; this is explained further in the methods section. Our analysis shows that both temporal and spatial resource partitioning contribute to coexistence, and highlights the role of seasonal fluctuations in biodiversity maintenance.

## Results and discussion

**Figure 1:**
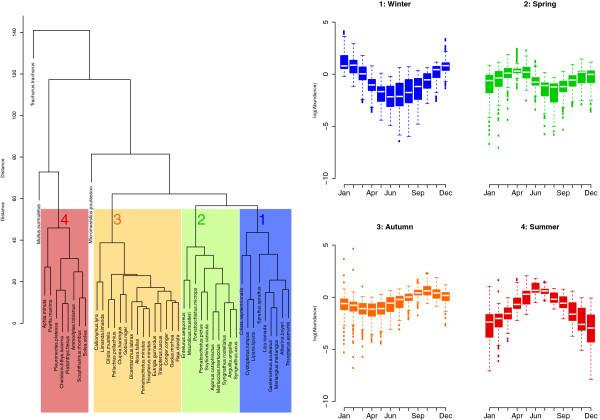
**Seasonal groupings in the fish assemblage at Hinkley Point.** Dendrogram: four seasonal groups of species identified by cluster analysis based on the seasonal fluctuation term in the model. This fluctuation term is driven by the water temperature and month effects, as identified in the fitted generalized additive models (GAMs). Box plots: the pattern of the ln-scaled relative abundances for each seasonal cluster: winter (group 1), spring (group 2), autumn (group 3) and summer (group 4).

Fish species at Hinkley Point fall into four seasonal groups (Figure 1), each group being abundant during winter, spring, autumn or summer (Figure 1, groups 1 to 4, respectively). Figure 1 illustrates the four clusters that emerge and shows the annual pattern of (ln) numerical abundance of the species in each cluster. There is nothing in the methodology used that predetermines the emergence of these four seasonal groups; rather the cluster analysis has captured the true temporal variation of the species in the data set. Other temporal trends would generate a different set or clusters, or even none at all; a randomization test (see Additional file 1) confirms this point.

**Figure 2:**
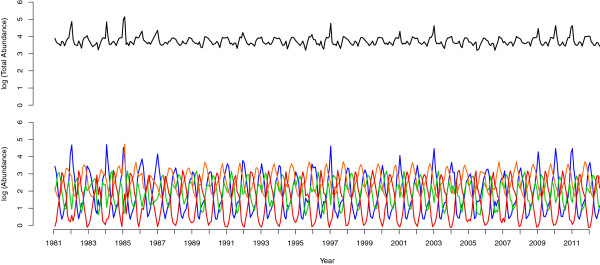
**Abundance of the community and the seasonal groupings through time.** Top: numerical abundance (ln) of the community through time. Bottom: the modeled seasonal component of the total relative abundance (ln) of the seasonal groupings shown in Figure 1 (winter (blue), spring (green), autumn (orange) and summer (red)) over the 31-year study.

The abundances of the four seasonal groups are offset throughout the 31-year study with the four seasonal groups ‘taking turns’ at being abundant (Figure 2). Interestingly, this pattern is maintained through the time series even though the abundance of each seasonal group (particularly winter) varies amongst years. While the total abundance of the community (Figure 2) also exhibits some seasonal and annual variation, this is muted relative to the variation within seasonal groups.

**Figure 3:**
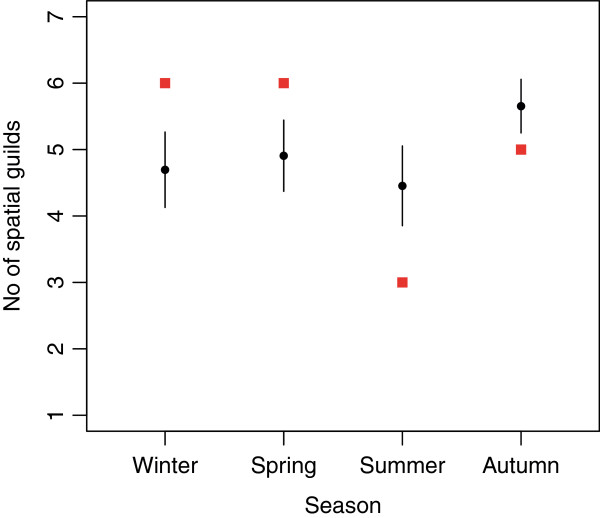
**Representation of spatial guilds in each of the seasonal groups.** The expected (black circles with 95% confidence intervals) and observed number (red squares) of different types of spatial guild in each seasonal group. The expected number of spatial guilds occupied in a given season was derived using a randomization test (see methods for details).

**Figure 4:**
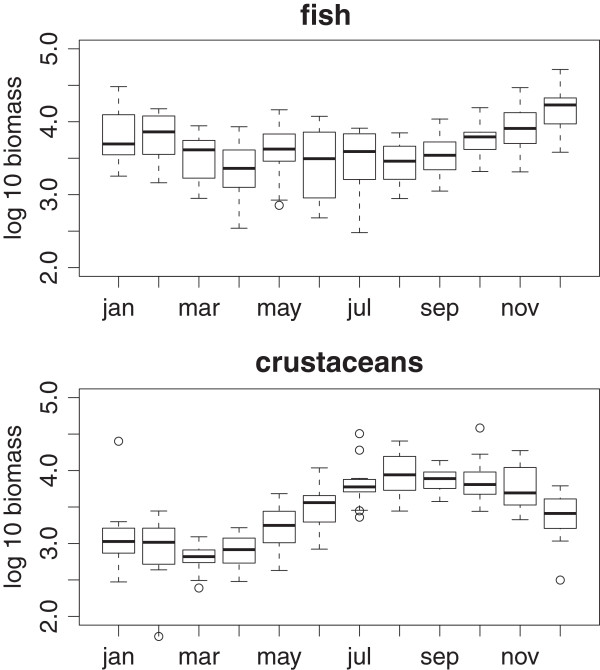
**Monthly variation in biomass.** Box plots for fish (top) and crustaceans (bottom) showing the monthly variation in total biomass (wet weight in g.).

**Figure 5:**
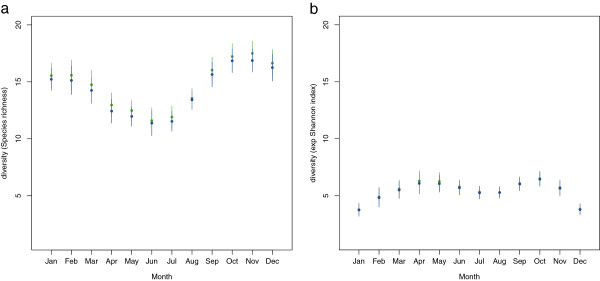
**Temporal trends in the diversity of the assemblage. (a)** The seasonal pattern of species richness (with 95% confidence limits) in the community. The plot shows the mean monthly values for both the core species (blue) and the entire community (green). **(b)** The seasonal pattern of diversity as measured by the exponential form of the Shannon index [34,35], again with 95% confidence limits, and shown for both the core (blue) and entire (green) community. As the Shannon index takes both evenness and richness into account the seasonal differences in the trends can be attributed to differences in evenness.

We used a randomization model to ask whether the number of spatial guilds exploited by each of the four species clusters identified is greater than what would be expected by chance. As Figure 3 reveals, this was the case for the winter and spring groups. In this system fish biomass peaks in the winter months (Figure 4). However, the biomass of crustaceans, an important component of the food web at Hinkley Point [33], is greatest in the summer and autumn (Figure 4). Indeed the general pattern in Figure 4 resembles classical predator–prey models, when predator abundance lags behind prey abundance. This suggests that the predators, that is, the fish, overshoot their prey, and experience more intense competition as a result. We have evidence, therefore, that the spatial segregation of species is most pronounced at those times when resources are most limiting. Interestingly, species richness (Figure 5a) shows a similar pattern to fish biomass, and lags behind peak crustacean biomass. In contrast, the assemblage becomes more even (as measured by the exponential form of the Shannon index (Figure 5b), which takes both evenness and richness into account) at the time when resources are most depleted.

Rosenzweig [32] argued that seasonal patterns of diversity deserve more attention. Our analysis vindicates his assertion. Taken together, the results show that the fish species in this assemblage fall into distinct seasonal groups. Two of the four temporal groups (winter and spring) are more diverse in terms of spatial guild occupancy than would be expected by chance if they were a random sample from the pool of species that inhabit the area. Because these are the times of year when competition for resources is likely to be greatest (Figure 4) this suggests that assemblage composition is driven by minimization of resource overlap via spatial and temporal segregation of species. In doing so, we provide support for the prediction, discussed by Wiens [36], Schoener [37] and others (see, for example, [38]) in the 1970s, that there will be increased spatial segregation at times when resources were limiting. These results are indicative of the link between community capacity [39] and species richness.

The patterns of temporal and spatial segregation we report are consistent with FMC predictions: species coexist by being abundant at different times and different places. Explicitly fitting FMC models to our core community of species would be intractable given the number of parameters involved when 45 species are interacting amongst themselves and with environmental variation. However, all the conditions for the storage effect are observed in this community. First, our model directly reports differences in species responses to the environmental variables (which correspond to the different coefficients in the model). Second, the seasonal changes in diversity are indicative of covariance between seasonal environmental variation and competition. Third, knowledge of the life history of these species tells us that although there is great variability, many species have larval stages and/or long lifespans, which buffer them from extinction at low abundance. Hence, the storage effect is likely to be operating in this community. Non-linearity is also likely because asymptotic resource acquisition curves are common among fish [40,41].

The asynchronous fluctuations we report have consequences for ecosystem functioning in the context of the biodiversity-stability debate. Since May [5] challenged the notion that species richness stabilizes ecosystems, ecologists have been trying to understand the relationship between diversity and stability. Experiments and theory point towards diversity increasing the magnitude of fluctuations in abundance of individual species, while stabilizing ecosystem-level properties [21,42]. In line with this, Figure 2 shows that fluctuations in total abundance are far less pronounced than those of the temporal groups. The mechanisms behind this pattern have been proposed to be: (1) differences in speed at which species respond to perturbations, (2) asynchrony in responses to environmental fluctuations, and (3) reduction in the strength of competition [43].

Our analysis is not directly relevant to the first mechanism, but knowledge of the system suggests it is likely to be observed. For example, the common eel, *Anguilla anguilla*, may take 20 years or more to complete its life cycle, whereas the transparent goby, *Aphia minuta*, is an annual fish that reproduces at 5 to 6 months old. These species are likely to differ markedly in response time to perturbations.

However, our model presents direct evidence that species respond differently to one key environmental variable, namely temperature, as this is the main driver of the seasonal change in species abundance. In addition, the responses of species to salinity and the North Atlantic Oscillation (NAO) also vary [44]. The analysis of spatial occupancy indicates that when resources are most limiting, species composition maximizes spatial guild dispersion. This results in minimized competition between species that co-occur temporally. In combination with temporal segregation, spatial segregation observed in this system is consistent with a spatiotemporal arrangement that minimizes competition. Thus, our results are indicative of the action of the last two of the proposed mechanisms of stabilization provided by biodiversity.

This paper has focused on the role of seasonality in promoting stability within the estuarine community. Longer-term environmental variation such as climate change, the NAO and even rainfall [45-48] also play a role in inducing turnover in species identity and abundance. Interestingly there has been no trend in measures of community structure, such as species richness, over the duration of the time series [44]. We suggest that these longer-term events work in tandem with seasonality to produce community stability through time.

Interest in phenological shifts has grown with the concern about climate change (see, for example, [2,3]). However, phenological studies often focus on population rather than community dynamics. Our investigation, together with those by Grøtan *et al.*[31] and Guo *et al.*[49], illustrate the need to consider the assemblage as a whole. If core species are abundant at different times, changes in their responses to seasonal drivers may have as yet unappreciated consequences for community responses to climate change. This point is underlined by recent work on the influence of seasonality on host-parasite systems [50] and on the links between functional traits and phytoplankton community structure [51].

## Conclusions

Our analysis shows that species segregate in space and time, and ‘take turns’ at being abundant in the community. Temporal fluctuation patterns are complex, but species cluster in seasonal temporal groups that peak in abundance at different times. Quantifying seasonal fluctuations in abundance helps explain how many species can coexist by not being simultaneously abundant.

## Methods

### Sampling methods

Fish have been sampled every month for 31 years from the cooling water filter screens at Hinkley Point ‘B’ power station, on the southern bank of the Bristol Channel in Somerset, UK (51°14’14.05’N, 3°8’49.71’W). The water intakes are in front of a rocky promontory within Bridgwater Bay, while to the east are the 40 km^2^ Steart mud flats.

Quantitative sampling commenced in 1980 when 24-h surveys of the diurnal pattern of capture were undertaken in October and November. From these surveys it was concluded that samples collected during daylight were representative of the 24-h catch, and monthly quantitative sampling commenced in January 1981. The total volume of water sampled per month, which has not varied over the 31-year period, is 4.27 × 10^5^ m^3^. Sampling represents a community over a 20 km length of coast [52]. To standardize for tidal influence, all sampling dates are chosen for tides halfway between springs and neaps, with sampling commencing at high water (normally about 12:00 pm). The number and species of fish and crustaceans collected hourly from two filter screens over a 6-h period are recorded. Monthly samples are taken over 6 h on an intermediate tide in the spring-neap cycle because the rate of capture of many animals varies with the tidal height, and a standardized sample covering the average tidal range is considered most suitable when calculating annual rates of capture. Depending upon the tide, the fish and crustaceans are sampled from water varying in depth from about 8 to 18 m. Fortunately, this sampling regime works well for most species and gives adequate sample sizes for even low abundance species.

The power station intakes at Hinkley Point are an effective sampler because of their location at the edge of a large intertidal mudflat in an estuary with extremely powerful tides, which generate suspended solid levels of up to 3 g/L, so that little light penetrates below 50 cm depth. Both pelagic and benthic fish are moved towards the intake in the tidal stream, often as they retreat from the intertidal zone where they feed. It is likely that they are unable to see or otherwise detect the intake until they are too close to make an escape. The filter screens have a solid square mesh of 10 mm and retain few fish less than 40 mm in length. The efficiency of the sampling method is discussed in Henderson and Holmes [47] and Henderson and Seaby [53]. Methodology has not changed over the entire study.

The wet weight of fish and crustaceans has been measured since 2000. We use this information to assess seasonal variation in resource limitation since the macrocrustaceans are an important component of the Hinkley Point food webs [33].

An important point to note is that the Bridgwater Bay habitat is a juvenile nursery. In essence, therefore we are recording abundance during a key early phase period. For many species, we are following them from about 3 months until about 2 years of age. Almost no fish breed in Bridgwater Bay so the majority of the species we study move elsewhere when adult to find a mate and lay their eggs.

### Statistical methods

To examine trends in numerical abundance we focus on the 45 core species that are consistently present in the assemblage [54]; the remaining 36 species occur infrequently and contribute only 0.1% of total abundance over the entire study. We begin by fitting a generalized additive model (GAM) [55] to the numerical abundance time series of each core species. Taking *Y*_*k*_(*t*) as a Poisson random variable representing the abundance of species *k* at time *t*, the model fit to the mean abundance E[*Y*_*k*_(*t*)] = *λ*_*k*_(*t*) is then given as:

(1) $$\begin{array}{lll} \log\left(\lambda_{k}\left(t\right)\right) & = & \beta_{0k}+s_{1k}\left(\text{Year}\right)+s_{2k}\left(\text{Tide}.\text{height}\right)\\ & & +s_{3k}\left(\text{Water}.\text{temp}\right)+s_{4k}\left(\text{Month}\right), \end{array}$$

Where the *β*_0*k*_ is a constant and *s*_*jk*_(·) is a smoothing spline function whose shape can be different over the factors, *j* = 1, 2, …, 4 as well as the species, *k* = 1, 2, …, 45. The equivalent degree of freedom for the smoothing splines is chosen to be 4 as default, which controls the smoothness of the functions, *s*_*jk*_, when they are estimated from the data. This model is decomposed into four components, each of which is driven by a different environmental factor namely year, tide height, water temperature and month. This additive form allows us to separate seasonal fluctuations from other nuisance components.

To investigate whether species are abundant at different times of year, in other words, how their seasonal patterns resemble one another, we assessed the extent to which the seasonal fluctuation in mean abundance is driven by the water temperature and month effects:

(2) $$\log\left(\lambda_{k}\left(t|s_{3},s_{4}\right)\right)=s_{3k}\left(\text{Water}.\text{temp}\right)+s_{4k}\left(\text{Month}\right).$$

The next step is to make groups of species based on the seasonal component (2) of the model. In other words, we identify species that show a similar seasonal pattern in their mean abundance, as modeled by the GAM. To do this we use hierarchical clustering that successively amalgamates groups of species on the basis of how similar they are in their seasonal pattern, using the distance measure described below. Importantly, there is no *a priori* assumption about the number of clusters to be made, nor of the distribution of the observed values (see Additional file 1); this is completely unsupervised clustering (R function: *hclust* is employed). We use Euclidean distance to construct the tree:

(3) $$d\left(j,k\right)=\sqrt{\sum_{t}{\left\{\log\left(\lambda_{j}\left(t|s_{3},s_{4}\right)\right)-\log\left(\lambda_{k}\left(t|s_{3},s_{4}\right)\right)\right\}}^{2}}$$

as a distance (or a dissimilarity index) between two species *j* and *k*, and the maximum distance between a pair of species, each of which belongs to a different cluster

(4) $$D\left(J,K\right)=\underset{j\in J;k\in K}{\text{max}}\;d\left(j,k\right)$$

as a distance between two clusters *J* and *K*.

Fish in this assemblage exploit a range of habitats with some species being associated with open water, others inhabiting rocky bottoms and so on. There are seven of these spatial guilds: the pelagic, proximo-benthic, hard-benthic, soft-benthic, weed and sheltered shallow guilds plus a group of migratory fish [56,57]. Fish in the different guilds exploit very different habitat types and are adapted to the conditions they find there. For example, fish that live in the pelagic zone, such as sprat (*Sprattus sprattus*) typically form large schools and have a fusiform body plan. In contrast species that are associated with the hard benthic zone including the conger eel (*Conger conger*) are often solitary and have a morphology that is suited to life amongst the nooks and crannies formed by rocks and stones. Flatfish such as plaice (*Pleuronectes platessa*) and sole (*Solea solea*) are associated with soft sediment. Spatial guild is thus a proxy for a set of traits linked to morphology and behavior. Fish may belong to different spatial guilds at different points of their lives. However, fish in the Hinkley Point community do not usually spend their entire lives in the estuary, and their membership of a spatial guild reflects their habitat use while they are present.

To minimize interspecific competition, the species within temporal groups should exploit available resources in different ways; we expect this effect to be strongest when competition for resources is greatest. If species segregate amongst spatial guilds due to limited resources, we should find that the seasonal groups are more diverse in terms of the spatial guilds represented than expected by chance. We calculate the random expectation as follows. Consider the case where *n* species are randomly chosen from a species list in which a total *m* species are classified into *G* different types of spatial guilds so each *g*-th guild have *m*_*g*_ species. Note that $m=\sum_{g=1}^{G}m_{g}$. Taking *Z* as the number of different types of guild found in such a random sample then the expected value and variance, respectively, are given as

(5) $$\begin{array}{ll} E\left[Z\right]= & G-\sum_{g=1}^{G}\left(\begin{array}{c} m-m_{g}\\ n \end{array}\right)\;{\left(\begin{array}{c} m\\ n \end{array}\right)}^{-1},\\ V\mathrm{ar}\left[Z\right] & =\sum_{g=1}^{G}\left(\begin{array}{c} m-m_{g}\\ n \end{array}\right)\;{\left(\begin{array}{c} m\\ n \end{array}\right)}^{-1}\left\{1-\left(\begin{array}{c} m-m_{g}\\ n \end{array}\right)\;{\left(\begin{array}{c} m\\ n \end{array}\right)}^{-1}\right\}\\ & \;+\;2\sum_{g<h}\left\{\left(\begin{array}{c} m-m_{g}-m_{h}\\ n \end{array}\right)\;{\left(\begin{array}{c} m\\ n \end{array}\right)}^{-1}\right)\\ & \;\left(-\left(\begin{array}{c} m-m_{g}\\ n \end{array}\right)\;\left(\begin{array}{c} m-m_{h}\\ n \end{array}\right)\;{\left(\begin{array}{c} m\\ n \end{array}\right)}^{-2}\right\}. \end{array}$$

## Competing interests

The authors declare that they have no competing interests.

## Authors’ contributions

AEM and MD designed the question, PAH developed the sampling design and collected the data, HS developed the analysis approach and analyzed the data. All authors contributed to writing the manuscript. All authors read and approved the final manuscript.

## Supplementary Material

Additional file 1 **Diversity is maintained by seasonal variation in species abundance.** Additional information concerning the cluster analysis of seasonal variation. Click here for file
