# Supplementary material for: Diversity is maintained by seasonal variation in species abundance
Source: BMC Biol. 2013 Sep 4;11:98. doi: 10.1186/1741-7007-11-98 (PMC3856667; doi:10.1186/1741-7007-11-98)
Supplement: Additional file 1 — Diversity is maintained by seasonal variation in species abundance. Additional information concerning the cluster analysis of seasonal variation. [file 1741-7007-11-98-S1.docx]

**Additional file for “ Diversity is maintained by seasonal variation in species abundance”**

Shimadzu, H., Dornelas, M., Henderson, P.A., Magurran, A.E.

The aim of this section is to demonstrate that the emergence of 4 seasonal groups is a characteristic of our data not an artifact of our analysis. We ran the following simulation test in which the same cluster analysis is applied to a set of completely random time series. We generate 45 random time series, each of which is regarded as each species’ numerical abundance, . As numerical abundances are discrete values, each series, , is generated from a Poisson distribution with a constant global mean, which is independent of time, , so that it is constant value over the time period. Since the variance of a Poisson distribution is equal to its mean, we need to fix only a mean value a priori for generating random time series. The constant mean for each species () is calculated from the data.

A GAM model is then fitted to each random time series, . The mean model in the logarithmic scale for each series is given as

where the is constant and is a smoothing spline whose shape can be different over the species, (). The seasonal component due to the random fluction,

is assessed to see whether any obvious seasonal fluctuation can be detected. The same simple hierarchical clustering approach is used to quantify the similarity of the seasonal components. This hierarchical clustering approach produces a set of clusters, successively amalgmating groups based on a distance measure described below; there is no a priori assumption about the number of clusters to be made, nor of the distribution of the observed values – this is completely unsupervised clustering (R function: *hclust* is employed). During the clustering process, each species starts in its own cluster, and pairs of clusters are merged as one moves up the hierarchy.We use Euclidean distance to construct the tree.

As can be seen in figure S1, this randomization test does not produce 4 seasonal groups, but rather results in one large cluster and another smaller one. The seasonal patterns exhibited by these groups differ markedly from the ones that emerged when the data were analysed (Figure S2). The simulation produced two clusters, one of which is very stable in abundance through time, the other exhibiting a more cyclical pattern of temporal abundance. This is very different from the analysis of the empirical data set in which the 4 temporal groups ‘take turns’ at being abundant.

**Figure S1 Dendrogram:** One large group, and one small group of species identified by cluster analysis based on the seasonal fluctuation term in the model. Box plots: the pattern of the log-scaled relative abundances for each cluster.

**Figure S2 Simulated abundance of the community and the cluster groupings through time**. *Top.* Numerical abundance (ln) of the community through time. *Bottom*. The modeled seasonal component of the total relative abundance (ln) of the two clusters of species.

**Identification of clusters**

Once a distance measure is chosen, the hierarchical clustering algorithm we used automatically produces a dendrogram, but not a set of clusters. Although there are no concrete guidelines for identifying the ‘true’ number of clusters, we have, in this study, used an objective method to determine the cutoff point that produces the most parsimonious set of clusters. This is described as follows:

Fig. S3a is a plot of the distance between the two clusters that are merged at each clustering step. The amalgamating process begins with each of the 45 species as a different cluster, and continues until there is one single cluster - the process chronologically progresses from the left to the right on the x-axis. The numbers (2-10) superimposed in Fig. S3a are the number of clusters remaining after each clustering step. It is clear that the distance between the two merged clusters increases monotonically towards the end of the process. The rate of the increase is largely dependent on the extent to which the clusters are distributed relative to one another. However, once the process reaches a point where the clusters are well segregated from each other, we expect the distance between the clusters to increase relative to that seen in previous steps; this is illustrated by the two different rates of increase in Fig. S3a. Accordingly, a change point of the increase is a good indicator of a meaningful number of clusters, in terms of how they segregate.

To identify a change point, we fit a piece-wise linear line (red line in Fig. S3a):

$$D_{s}=\left\{\begin{matrix} a_{0}+a_{1}s+e_{s}, & if s<s* \\ b_{0}+b_{1}(s-s*)+e_{s}, & if s\geq s* \end{matrix}\right.$$

where $D_{s}$ is the distance between the two clusters merged at the $s$-th clustering step and $s*$ is the change point that is estimated with the other parameters, $a_{0}, a_{1}, b_{0}$and $b_{1}$, by minimizing the sum of squared residuals, $\sum_{s} e_{s}^{2}$. Here, the coefficients $a_{1}$ and $b_{1}$ are the two different rates of the increase. Fig. S3b shows the sum of squared residuals at different change points, and indicates that $s*=39$, at which the number of clusters is seven, is the optimal change point in terms of minimizing the sum of squared residuals. We have therefore chosen our clusters to be these seven clusters - the four seasonal groups and three singletons (Fig. S3b, Fig. S4).

**Figure S3a and b.** Identification of change point in cluster analysis. The numbers of the graph refer to the points at which the clusters are merged (as indicated in Fig. 2 below)

**Figure S4.** The clusters represented by the change points identified in Fig 1a and b.
